# Impact of Anesthesia Protocols on *In Vivo* Bioluminescent Bacteria Imaging Results

**DOI:** 10.1371/journal.pone.0134048

**Published:** 2015-07-24

**Authors:** Thomas Chuzel, Violette Sanchez, Marc Vandamme, Stéphane Martin, Odile Flety, Aurélie Pager, Christophe Chabanel, Luc Magnier, Marie Foskolos, Océane Petit, Bachra Rokbi, Emmanuel Chereul

**Affiliations:** 1 Voxcan, Marcy-l’Etoile, France; 2 Sanofi Pasteur, Campus Mérieux, Marcy-l’Etoile, France; Cornell University, UNITED STATES

## Abstract

Infectious murine models greatly benefit from optical imaging using bioluminescent bacteria to non-invasively and repeatedly follow *in vivo* bacterial infection. In this context, one of the most critical parameters is the bioluminescence sensitivity to reliably detect the smallest number of bacteria. Another critical point is the anesthetic approaches that have been demonstrated to impact the bioluminescence flux emission in studies with luciferase-transfected tumor cells. However, this impact has never been assessed on bacteria bioluminescent models. To this end, we investigated the effects of four anesthesia protocols on the bioluminescence flux in a central venous catheter murine model (SKH1-hr^hr^ mice) infected by a bioluminescent *S*. *aureus* Xen36 strain. Bioluminescence imaging was performed on mice anesthetized by either ketamine/xylazine (with or without oxygen supplementation), or isoflurane carried with air or oxygen. Total flux emission was determined *in vivo* daily for 3 days and *ex vivo* at the end of the study together with a CFU counting of the biofilm in the catheter. Bioluminescence flux differences appear between the different anesthetic protocols. Using a ketamine/xylazine anesthesia (with air), bacteria detection was impossible since the bioluminescence signal remains in the background signal. Mice anesthetized with isoflurane and oxygen led to a signal significantly higher to the background all along the kinetics. The use of isoflurane in air presents a bioluminescence signal similar to the use of ketamine/xylazine with oxygen. These data highlight the importance of oxygen to improve bioluminescence flux by bacteria with isoflurane as well as with ketamine/xylazine anesthetics. As a conclusion, we recommend the use of isoflurane anesthetic with oxygen to increase the bioluminescence sensitivity in this kind of study.

## Introduction

For more than a decade, *in vivo* bioluminescence imaging (BLI) has become an increasingly used approach to follow non-invasively the development and the evolution of complex processes in *in vivo* models. A huge number of pathologies were mimicked through animal models in which physico-pathological pathways were labeled by luciferase as a reporter gene. Numerous models were also created in the oncology field by injection of cancer cell lines transfected with the luciferase gene. Several bacterial strains were rendered bioluminescent through the stable integration of an unaltered *P*. *luminescense luxCDABE* (for Gram-negative bacteria [1,2]) or an optimized *P*. *luminescense luxABCDE* (for Gram-positive *bacteria* [2–4]) cassette into their chromosome to *in vivo* investigate bacterial infection models. Performing bioluminescent imaging of physiological processes in transgenic animals or oncological rodent models requires the injection of luciferase’s substrate, the D-luciferin which, in the presence of the enzyme, oxygen and ATP, generates visible light. In contrast, for bacterial infection models, no additional injection of exogenous product is needed. Indeed, all bacteria used for bioluminescent studies were transformed so that an optimized *Photorabdus luminescence lux* operon is integrated into their chromosome which encodes the genes required for both luciferase and substrate production.

For the first two models requiring the luciferase’s substrate injection, generally through intraperitoneal route, D-luciferin bio availability appeared to be a major parameter for the model validation. It is widely explained by optical imaging system providers, and well known by the users of this technology, that light emission increases gradually in the minutes following the injection to achieve a maximum of intensity. This maximum is followed by a plateau (equilibrium state between all the components of the enzymatic reaction) before a decrease of the light intensity, corresponding to the consumption of D luciferin. Durations of the initial increase of light intensity and the plateau phase, as well as the maximum light intensity, are parameters tightly correlated to the availability of both D-luciferin and oxygen in the vicinity of the enzyme.

Recent studies have shown the impact of anesthetics methods on bioluminescence total flux emission by cells in transfected tumor cells studies [5,6]. To our knowledge, this kind of study has never been reported on bacterial infection models. The aim of this study was to investigate the effect of four different anesthesia approaches on *in vivo* bioluminescent emission in a CVC (Central Venous Catheter) murine model mimicking nosocomial infection [7–9]. To this end, the effects of ketamine/xylazine with or without supplementation of oxygen or isoflurane carried with air or oxygen were compared to determine the effects of anesthesia protocol on bacteria detection and bioluminescence total flux emission.

## Materials and Methods

### Ethics statement

All experiments were performed in accordance with national animal care guidelines (EC directive 2010/63/UE, French decree n° 2013–118) and study was approved by the local ethics committees (VetAgro-Sup), authorization number n°1413.

### Bioluminescent bacteria

The commercial gram positive bioluminescent *Staphylococcus aureus* Xen36 strain was used (Bioware, Perkin Elmer) in this study. *S*. *aureus* Xen36 is derived from the parental strain *S*. *aureus* ATCC *49525* (Wright), a clinical isolate from a bacteremia patient. This light producing microorganism possesses a stable copy of the modified *Photorhabdus luminescens luxABCDE* operon (3) at a single integration site on a native plasmid. This bacteria has both genes encoding luciferase and luciferin, thus no injection of exogenous product is needed to obtain the bioluminescence.

### Preparation of *S*. *aureus* strain for mice challenge

Bacteria were grown onto tryptic soy agar (TSA, BioMerieux) at 37°C overnight. The challenge suspensions were obtained from a subculture in tryptic soy broth (TSB, Difco) at a 0.2 optical density estimated by measuring the 680 nm absorbance of the overnight culture. Then bacteria were grown at 37°C for 2 hours. Bacteria were pelleted, resuspended and washed once in PBS. Bacterial inocula of *S*. *aureus* Xen36 were adjusted at 2.5x10^8^ colony forming units (CFU)/mL. CFUs of the suspensions before and after the inoculation in mice were verified after overnight culture.

### Mouse model

In order to evaluate impact of the anaesthesia procedures on bacteria bioluminescence follow-up, a model mimicking the human conditions of iatrogenic infections induced by medical devices was used. To this end, a specific Central Venous Catheter (CVC) mouse model was developed using 8 weeks old immunocompetent male hairless SKH1-hr^hr^ (Crl:SKH1-hr) mice, purchased from Charles River (France) and acclimated 2 weeks before experimentation. Experiments were performed in a Biosafety level 2 animal unit and laboratory (ABSL-2); and animals were housed collectively on sawdust (Toplite-Select Fine, Safe) in standard filtered cages inside a controlled ventilated rack (XJ Mouse rack, Allentown USA) under a 12h day/night cycle with free access to tap water and food *ad libidum* (A04 diet, Safe). During all the experimentation, mice were monitored at least once a day and all events were recorded.

Briefly, a single lumen sterilized fluorinated ethylene propylene catheter (Catheter Surflo-W 26G, Terumo, 0.45/0.65 mm internal/external diameters) was surgically placed in the right jugular vein of mice previously anesthetized with 2% isoflurane in air (Aerrane, Baxter, USA). A subcutaneous injection of Buprenorphine (BUPRECARE 0,3mg/ml, AXIENCE) at 0.05 mg/kg was performed before surgical procedure and twice a day for two days.

After one week of recovery, mice were anesthetized according to their protocol groups (see below) prior to challenge by intravenous (IV) injection in the tail vein with a 3.3x10^7^ CFU dose of *S*. *aureus* Xen36 strain (200μL/mouse). At the end of experimentation, all mice were anesthetized according to their protocol group and then sacrificed by an intra-cardiac injection of Dolethal (Vetoquinol).

### Anesthesia protocols

We evaluated the impact of four different anaesthesia protocols on bacteria bioluminescence in our CVC model. To this end, catheterized mice were randomly assigned in four anesthesia groups of 10 mice before challenge with the *S*. *aureus* Xen36 strain. The first protocol consists of an intraperitoneal injection of a mix of ketamine (Imalgene 1000, Merial, 100 mg/kg) and xylazine (Rompun 2%, Bayer, 8 mg/kg) (hereafter K&X). The second is the same anesthetic injection with an oxygen supply (100% O_2_, ALSF-097A, Air Liquide Healthcare) by a respiratory mask just after injection and during all the imaging acquisition (hereafter K&X+O_2_). The third anesthesia protocol is a gaseous induction with Isoflurane (2%—Aerrane, Baxter, USA) in air as a carrier gas (ISO+air) and the last anesthesia protocol is a gaseous induction with 2% Isoflurane in oxygen as a carrier gas (hereafter ISO+O_2_).

### Bioluminescence acquisition

To follow bacterial growth, a longitudinal whole-body non-invasive optical imaging was performed on anesthetized mice at D0, D1, D2 and D3. After the last *in vivo* acquisition, mice were euthanized and an *ex vivo* bioluminescence imaging was performed on catheters before determining biofilm CFU number (detailed below).

Bioluminescence imaging follow-up was performed with an IVIS Spectrum (Perkin-Elmer). Anesthetized mice were positioned at each acquisition in dorsal decubitus on a heated platform (37°C) in order to maintain their physiological body temperature. The photon emission expressed in total flux (photons/second, [p/s]) was measured by a high sensitivity CCD camera during 3 minutes in a square Field of View (FOV) of 13.1 cm of side. Images were obtained with a binning parameter of 16 and an F/stop parameter of 1 (diaphragm opened at maximum). After the last *in vivo* bioluminescence imaging, mice were sacrificed and catheters were withdrawn and positioned in an uncovered petri dish. Acquisition parameters for *ex vivo* catheters were similar to the *in vivo* ones.

**Fig 1:**
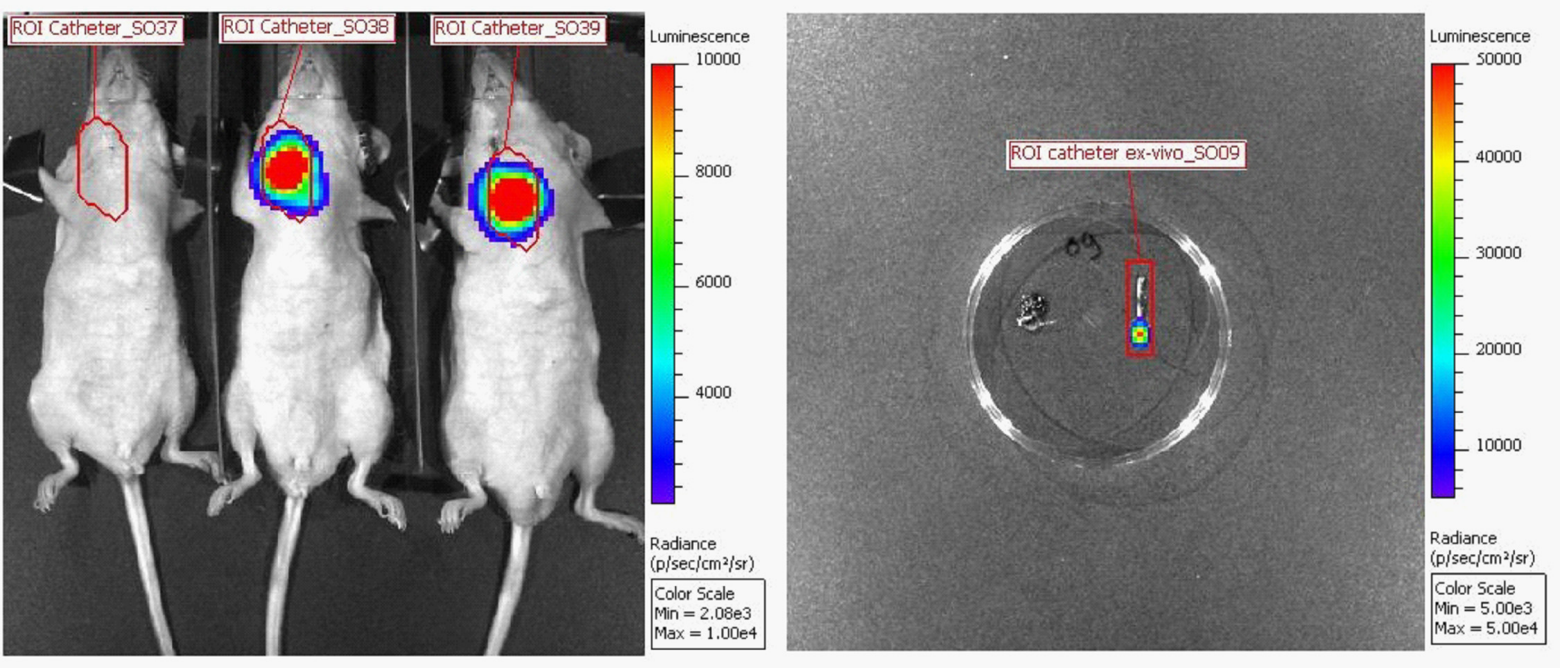
Bioluminescence images obtained *in vivo* (left) and *ex vivo* (right) with ROIs delineated around each catheter.

A Region Of Interest (ROI) was delineated with the software “Living Image”, version 4.4 (Perkin Elmer), around the catheter of each mouse (**Fig 1**). Each ROI always had the same area. Three operated mice called “Background” received only an injection of PBS instead of bacteria on D0 in order to obtain the background photon level on non-infected mice. These mice allow the calculation of the mean total flux background value, which represent the flux naturally emitted by the mice plus the machine electronic noise. This value is mentioned directly on the total flux results.

### Quantification of Adherent Bacteria on the catheters

After *ex vivo* imaging, catheters were immerged in 10 mL of phosphate buffered saline (PBS) 0.5% Tween 20 (Prolabo) at +4°C. Any residual tissue or suture threads were removed from catheters in sterile Petri dish. Catheters were transferred in sterile tubes and washed twice with 10 mL of PBS 0.5% Tween 20 to remove any non-adherent bacteria from their surface. Bacteria were then detached from the catheters by two sonications in 1 mL of PBS 0.5% Tween 20 for 15 min at 35 kHz (ultrasonic cleaner VWR) followed by agitation with vortex during 15 seconds (Top Mix Fisher Scientific).

For quantification of adherent bacteria, an aliquot of the suspension was serially diluted and spread in six drops on agar plates (Trypticase Soy Agar; Biomerieux). After incubation for 18 to 24 hours, CFU were counted for each plate and averaged for each group.

### Data presentation and statistical analysis

All data are represented as mean +/- Pearson SD. Statistical analysis was performed with the GraphPad Prism v.6.0 software. *In vivo* and *ex vivo* mean total fluxes of each group at each time point were compared using a non-parametric test (Mann and Whitney). A p-value < 0.05 was considered statistically significant. The degree of statistical significance of p-result are mentioned directly in the results: * < 0.05.

## Results

### Impact of anesthesia methods on *in vivo* bioluminescence total flux

**Fig 2:**
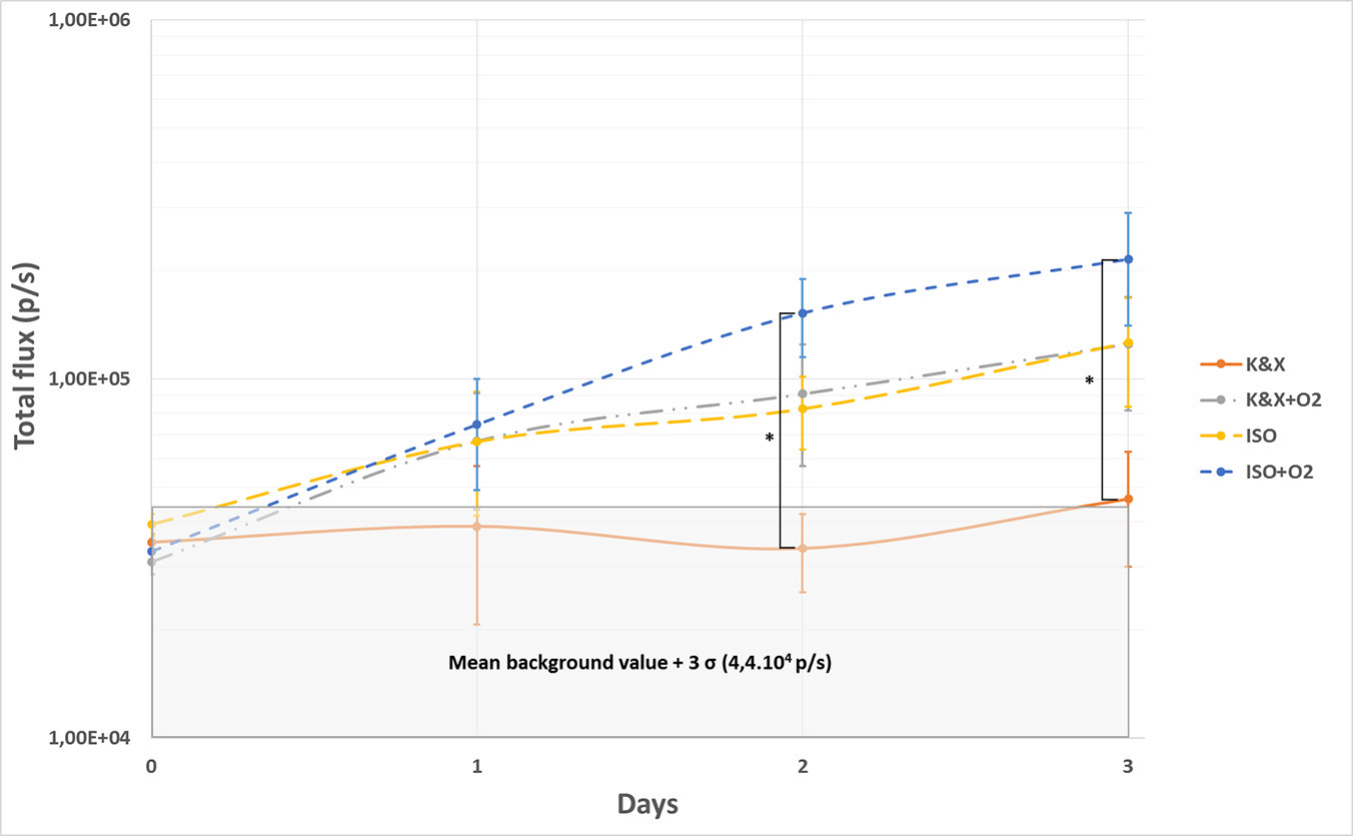
Mean total flux at the catheter localization obtained *in vivo* from D0 to D3 for the four anesthesia protocols. Statistically significant differences were observed between two groups (K&X vs ISO+ O_2_) on D2 and D3 (*p* < 0.05).

Impact of the anesthesia methods in our CVC infection mouse model was assessed for 3 consecutive days. At each bioluminescence imaging session, mice were anesthetized according to their group protocol and photon emission by the mouse was recorded and reported as a bioluminescent flux in photons/second (p/s). At D0, a similar bioluminescence flux at the catheter localization was observed, just after IV infection in all groups (**Fig 2**). In K&X group, the bioluminescence signal was stable along the study and did not differ from the background signal determined in three operated but not infected mice. In contrast, we observed an increase of bioluminescence signal outside the background signal for the three other anesthesia protocols evaluated. K&X+O2 and ISO+air groups presented similar bioluminescence values from D0 to D3. This signal increased 1.7, 2.9 and 2.7 times at D1, D2 and D3 respectively between K&X and K&X+O2 groups (no significant differences). Moreover, addition of oxygen to isoflurane protocol leads to an increase of bioluminescence signal as compared to ISO+air group. This signal increased 1.8 and 1.7 times at D2 and D3, respectively. Indeed, bioluminescence total flux in this group was five times higher than background and K&X groups signal with significant differences observed between K&X and ISO+O2 groups at D2 and D3.

### Impact of anesthesia method on *ex vivo* bioluminescence total flux

**Fig 3:**
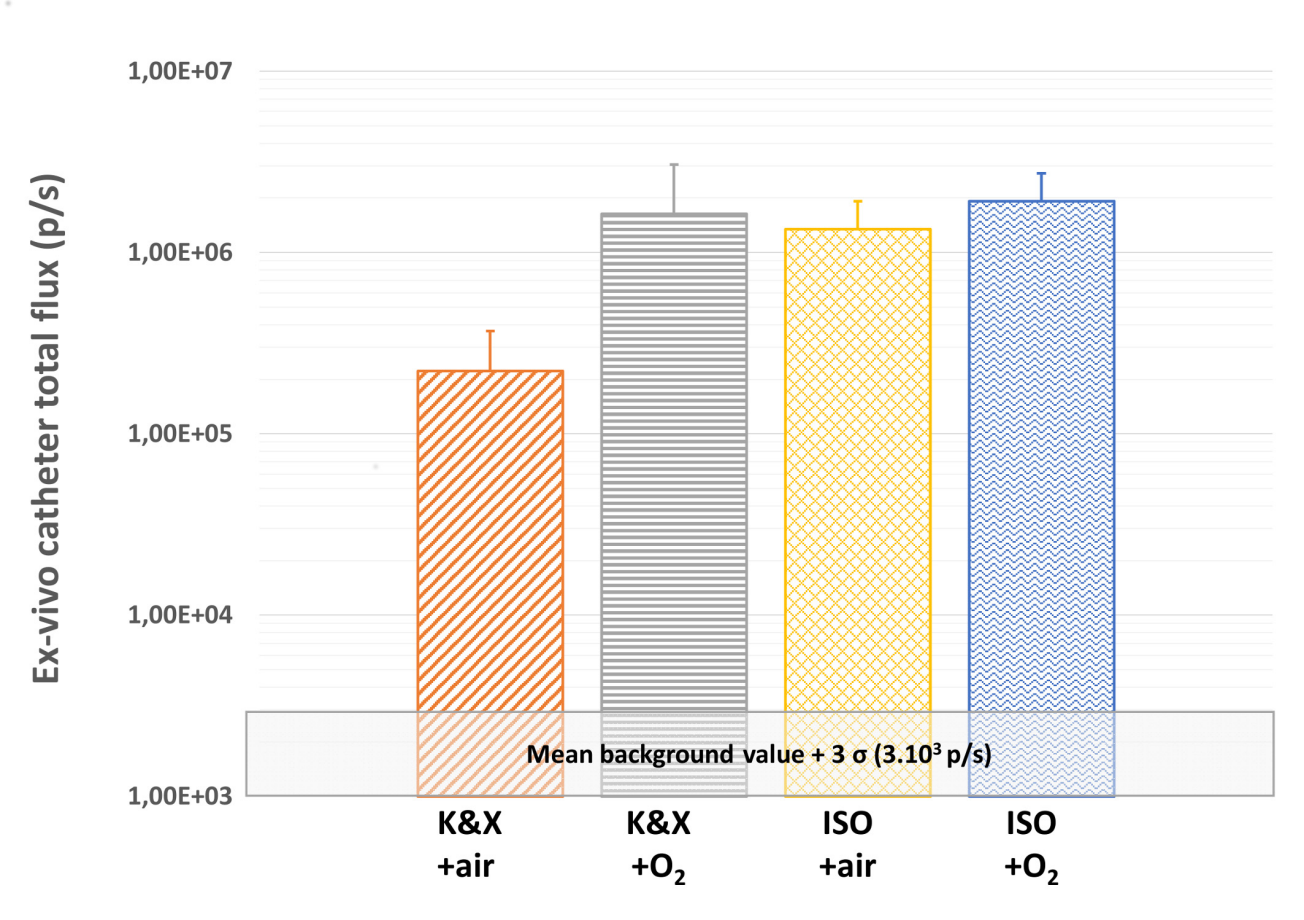
Mean bioluminescence total fluxes obtained *ex vivo* on catheters just after sampling for the different anesthetics protocol. Statistical analysis exhibited no significant differences between groups.

At the end of bioluminescence follow-up (D3), an *ex vivo* bioluminescence imaging was performed after catheter sampling. Whatever the anesthesia protocol considered, the mean bioluminescence signal in the different groups was above the background signal (**Fig 3**). Similar bioluminescence signals were observed *ex vivo* in groups K&X+O_2_, ISO and ISO+O2, whereas a ~7 times non-significant lower level was obtained in K&X group.

### Impact of anesthesia method on CFU number

**Fig 4:**
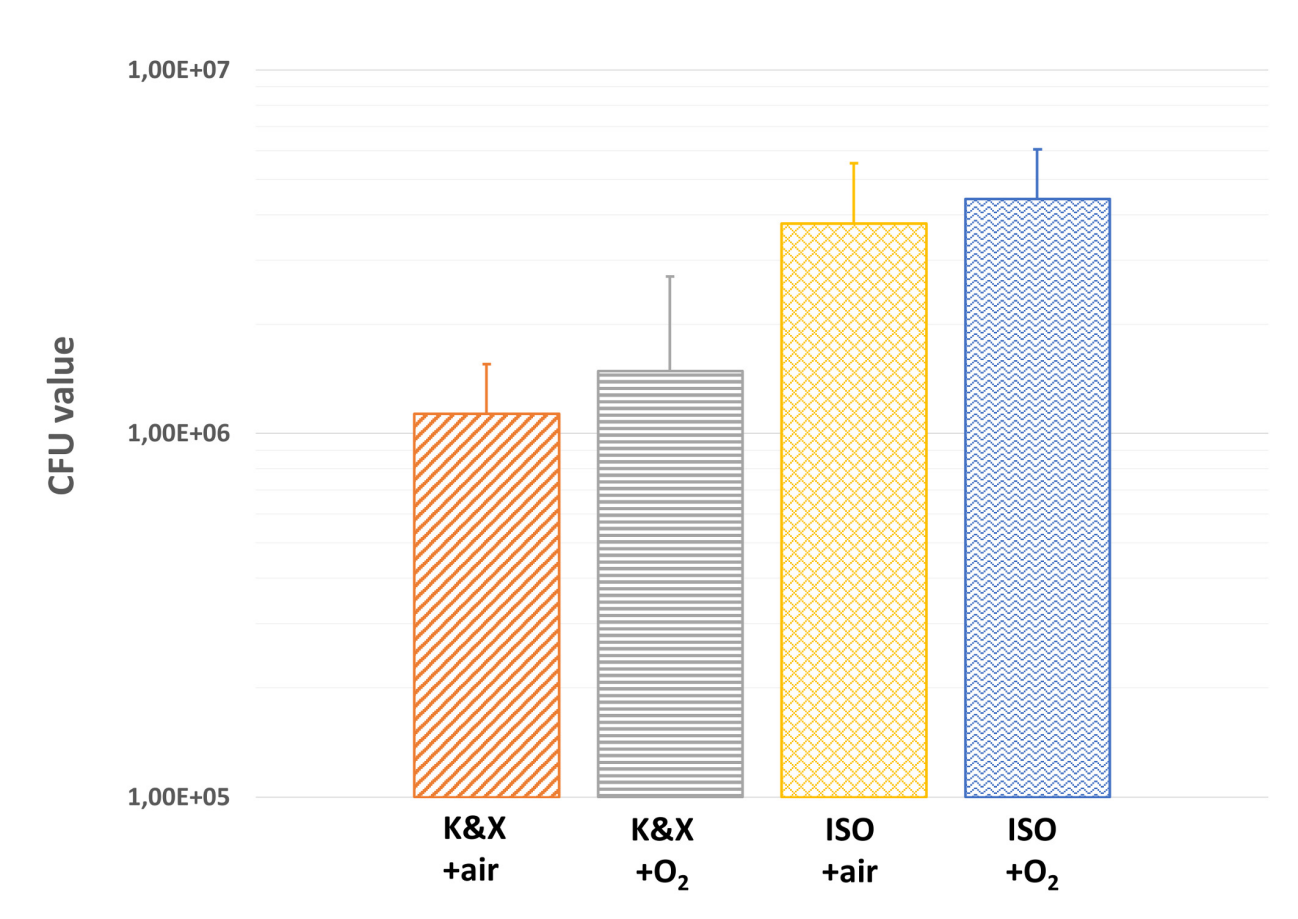
Mean CFU values obtained ex *vivo* on catheters after counting on D3 for K&X, K&X+O_2_, ISO and ISO+O_2_. Statistical analysis exhibited no significant differences between groups.

To assess the effect of anesthesia protocol on bacteria growth, a CFU counting of adherent bacteria inside the catheter was performed at D3. The number of infected catheters was addressed in each anesthesia condition: 3 catheters were free of bacteria in both K&X and K&X+O_2_ groups, while 2 catheters in ISO group and only 1 catheter in ISO+O_2_ group were bacteria free. A similar CFU number (~1.5x10^6^ CFU) was observed in groups anesthetized with K&K or K&K+O_2._ The use of isoflurane containing protocols induced a 4 times increase of CFU number as compared to K&X protocols. We did not observe any difference in CFU number between ISO+air and ISO+O2 groups (**Fig 4**).

## Discussion

In this study, we investigated the effects of four different anesthesia protocols both on the total bioluminescence flux and the number of luminescent bacteria. The use of such bacteria allows a non-invasive real-time and repeated monitoring of the bacterial infection *in vivo*, enhancing their potential for testing vaccine candidates or evaluating therapeutic approaches for the prevention and treatment of infection [10–12]. In this context, the total bioluminescence flux is an important parameter, a high luminescence efficiency lead to a better sensitivity and thus the possibility to detect a small number of bacteria *in vivo*. In this view and regarding the increasing number of studies using quantitative bioluminescence techniques, an important parameter which could affect total flux is the choice of anesthetic procedure [6]. Indeed, recent publications reports the impact of anesthetic conditions on BLI quantification in tumor follow-up studies using transfected luciferase tumor cells with a clear inhibitory effect of volatile anesthetics on the luciferase activity [5,13]. To our knowledge, this type of evaluation has never been performed using bacterial luciferase systems (operon lux) in *in vivo* studies.

To evaluate the most frequently used anesthesia protocols, mice were implanted with a central venous catheter followed few days later by a systemic i.v bacterial inoculation with *S*. *aureus* Xen36. The catheter site was then imaged daily for three days. We demonstrated a significant difference in bioluminescence fluxes between anesthetic conditions. The use of a ketamine/xylazine anesthesia alone leads to a poor infectious model regarding both the total flux emission and the number of infected catheter and with the lowest BLI and the lowest CFU counting. On the contrary, the use of isoflurane carried in oxygen allows the most sensitive follow-up of bacteria growth at the catheter level all along the study with the best detection of BLI and the highest CFU counting.

According to the literature, the discrepancy of bioluminescence fluxes could be explained by various factors, including substrate and oxygen availabilities, bacteria metabolic activity or signal localization (background, diffusion…) [6]. In our context, most of these parameters can be ruled out, since mice have experienced the same surgical procedure and were challenged in the same conditions. Moreover, the substrate bioavailability can also be excluded, since bacteria used in this study has a gene construction encoding for both luciferase and the substrate (operon lux). The only differences between the four groups of our study were the nature of the anesthetics used and the potential addition of oxygen.

Among other factors affecting the bioluminescence flux, oxygen is a well-known parameter which can potentially impact photon emission by the microorganism considering the dependence of luciferase reaction with oxygen availability [14]. To identify the importance of oxygen in our study, each anesthetic was used with and without an exogenous supply of oxygen. Although not statistically significant, we found the addition of O_2_ to K&X and ISO led to an increase of BLI emission by bacteria. In our case, the CFU number determined in K&X group showed the presence of adherent bacteria inside the catheter in a group where we have observed no significant bioluminescence *in vivo* as compared to the background and the lowest *ex vivo* flux as compared to others groups. These data suggest K&X has an inhibitory effect on bacterial bioluminescence. Indeed, anesthetics have been recognized to interact at the molecular level with luciferase enzyme resulting in a decrease of luminescence emission [15]. Moreover, the CFU number determined at D3 showed a smaller number of adherent bacteria in K&X than in ISO groups. For both anesthetics, the supply of oxygen did not modify the number of adherent bacteria on the catheter. Further supporting this hypothesis of a direct toxic effect of anesthetics repeated injection, the number of infected catheters is lower in K&X groups than in ISO groups (respectively by mean 64% versus 85%). The impact of anesthetics should also be considered at the organism level, mainly the consequences on the cardiovascular system [16,17]. In a comparison study, isoflurane resulted in the smallest reduction of cardiac output. Isoflurane anesthesia preserves cardiac function better than other anesthetic regimens and preserves peripheral organ perfusion [16]. The strongest impact on cardiac output and heart rate is expected under K&X anesthesia, which induces hypotension and hypothermia [16,18].

These parameters will have also a potential impact of oxygen bioavailability in the vicinity of bacteria impacting both luminescence efficiency and bacterial growth with ketamine/xylazine. Moreover, the duration of anesthesia is also longer under ketamine/xylazine anesthesia than isoflurane and both ketamine/xylazine molecules remains in organisms for hours [19]. Based on these data, a repeated bioluminescence imaging each day may increase the impact of ketamine/xylazine on bacteria.

## Conclusion

This study sheds light on the particular attention we should pay to anesthesia when performing *in vivo* studies on infection rodent models using bioluminescent bacteria. The use of isoflurane and oxygen as a gas carrier allowed the follow-up of bioluminescent bacteria with the greatest sensitivity, particularly in comparison with ketamine/xylazine protocols. Moreover, a trend showing a deleterious effect of ketamine/xylazine both on bacteria luminescence efficiency and on bacteria growth was observed. Based on our data, we strongly recommend the use of isoflurane with oxygen to increase the sensitivity in bioluminescent bacteria model studies.
